# SOX5 promotes cell growth and migration through modulating DNMT1/p21 pathway in bladder cancer: SOX5/DNMT1/p21 pathway in BC

**DOI:** 10.3724/abbs.2022075

**Published:** 2022-06-21

**Authors:** Longxiang Wu, Zhongqing Yang, Guoyu Dai, Benyi Fan, Junbin Yuan, Yalin Liu, Peihua Liu, Zhenyu Ou

**Affiliations:** 1 Department of Urology Xiangya Hospital Central South University Changsha 410008 China; 2 National Clinical Research Center for Geriatric Disorders Xiangya Hospital Central South University Changsha 410008 China; 3 Xiangya School of Medicine Central South University Changsha 410008 China

**Keywords:** bladder cancer, SOX5, DNA methylation, DNMT1, p21

## Abstract

Bladder cancer (BC) is one of the most prevalent and life-threatening cancers among the male population worldwide. Sex determining region Y-box protein 5 (SOX5) plays important roles in a variety of human cancers. However, little research has been conducted on the function and underlying mechanism of SOX5 in BC. In the present study, we first reveal the increased expression of SOX5 in BC tissues and *in vitro* cells lines. Second, we discover that inhibition of SOX5 inhibits cell growth and migration but promotes cell apoptosis. Meanwhile, ectopic SOX5 expression stimulates cell growth and migration in BC cells. Then, we show that suppressing SOX5 inhibits the expression of DNA methyltransferase 1 (DNMT1), and that overexpressing DNMT1 alleviates the cell progress of BC cells inhibited by SOX5. Furthermore, we demonstrate that DNMT1 inhibits p21 expression by affecting DNA methylation of the p21 promoter. Collectively, we demonstrate that SOX5 exerts its functions in BC cells by modulating the SOX5/DNMT1/p21 pathway. Finally, we demonstrate that SOX5 knockdown inhibits xenograft tumor growth *in vivo*. In conclusion, our study elucidates the oncogenic role of SOX5 and its underlying molecular mechanism in BC, and reveals a novel pathway which has the potential to serve as a diagnostic biomarker and therapeutic target for BC.

## Introduction

Bladder cancer (BC) is one of the most prevalent and life-threatening cancers among the male population worldwide [1]. Currently, BC has the highest incidence of urinary system tumors and has a significantly higher mortality rate in China [2]. Despite significant advances in diagnosis and treatment, the recurrence rate of BC patients remains around 75%, resulting in a low level of five-year overall survival rate [3]. Furthermore, the biomarkers and molecular mechanisms underlying the development and progression of BC need to be investigated further.

SOX5, a transcription factor in the Sex determining region Y-box (SOX) family, is involved in a variety of cellular processes, including cell growth, cell apoptosis, cell cycle, cellular senescence, aging, and metabolism [4]. Furthermore, SOX5 has been linked to physiological and pathological processes in a variety of cancers, including breast cancer, gastric cancer, lung cancer, ovarian cancer, colorectal cancer, and hepatocellular carcinoma [ 5– 7] . Previously, we discovered that silencing of *SOX5* inhibited cell progress in BC [8]. However, the biological roles and the precise underlying regulatory mechanism of SOX5 in BC remain unknown.

In the process of DNA methylation, a methyl group from S-adenosylmethionine is transferred to the C-5 position of cytosines residing in CpG dinucleotides [9]. In mammals, three types of DNA methyltransferases (DNMTs), *i*. *e*., DNMT1, DNMT3a, and DNMT3b, catalyze DNA methylation [10]. The most abundant DNMT is DNMT1 which maintains DNA methylation patterns in newly synthesized DNA, whereas DNMT3a and DNMT3b methylate fully unmethylated CpG sites [ 11, 12] . DNA methylation has an impact on a variety of cellular processes, including chromosome stability, chromatin structure, genomic imprinting, transcription, X chromosome inactivation, embryonic development, cell growth and death, and so on [12]. DNA methylation plays a significant role in the physiological and pathological progression of human diseases such as imprinting disorders, repeat-instability diseases, and cancers [13]. Many cancers have been shown to have oncogenic DNMT1-mediated DNA methylation [14]. DNMT1 has been linked to the physiological and pathological progression of BC [ 15, 16] . Nevertheless, the upstream and downstream regulatory factors and mechanisms of DNMT1 in BC require further investigation.

As a downstream target of the well-known tumor suppressor p53, the cyclin-dependent inhibitor p21 inhibits cell growth and migration but promotes cell apoptosis and cell senescence [17]. Several studies focused on the inhibition of p21 expression by DNMT1-mediated DNA methylation [ 18, 19] . Another study focused on the role of p21 in the regulation of DNMT1 expression [20]. However, the relationship between DNMT1 and p21 in BC has not been studied.

Based on previous studies, we focused on investigating the function and the underlying mechanism of SOX5 in BC. We demonstrated that increased SOX5 expression in BC promotes DNMT1 expression, resulting in p21 inhibitory expression and, ultimately, the development and progression of BC.

## Materials and Methods

### Clinical specimens

All tumor tissue samples and adjacent normal tissues (located > 5 cm from tumors) were collected under the protocols approved and supervised by the Research Ethics Committee of the Xiangya Hospital, Central South University (Ethics Approval number: 202103548). All the tissues were immediately snap-frozen in liquid nitrogen. Informed consent agreements (written in the light of the Ethical Guidelines) were obtained from all patients. Patients’ clinical information was listed in Table 1.

**Table:** **Table 1** Clinical information of BC patients

Characteristics	Age	Gender	T stage	N stage	M stage	Grade
	≤65	>65	Male	Female	Ta–T1	T2–T4	N	N+	M–	M+	High	Low
Number	24	8	31	1	14	18	27	5	32	0	25	7

### Cell culture

The human BC cell lines (5673, J82, T24 and EJ-M3) and bladder epithelial cells (SVHUC-1) were purchased from the American Type Culture Collection (ATCC; Manassas, USA) and routinely preserved in our laboratory. All the cells were maintained in Dulbecco’s modified Eagle’s medium (DMEM; Gibco, Carlsbad, USA) supplemented with 10% fetal bovine serum (FBS; Gibco), 1 ×penicillin/streptomycin (Gibco), and cultured at 37°C in a humidified cabinet with 5% CO _2_.

### Treatment with 5-aza-2’-deoxycytidine

The 5-aza-2’-deoxycytidine (5-AzadC) was obtained from Sigma-Aldrich (St Louis, USA) and dissolved in dimethyl sulfoxide (DMSO; Sangon Biotech, Shanghai, China). 5-AzadC was added into the cells and incubated for 24 h at a concentration gradients of 0, 0.5, 1.0 and 2.0 μM.

### Small interfering RNA (siRNA), plasmid construction and transfection

SiRNAs against SOX5 (SOX5-siRNAs), DNMT1 (DNMT1-siRNAs), p21 (p21-siRNAs) and negative control siRNA (NC-siRNAs) were synthesized by Genepharma (Shanghai, China), and the sequences were shown in Table 2.

**Table:** **Table 2** The sequences of siRNAs used in the study

Name	Sequence (5′→3′)
siSOX5-1	sense	GACCAUGAUGCUGUCACCAAGGCAA
antisense	UUGCCUUGGUGACAGCAUCAUGGUC
siSOX5-2	sense	GAGAAGUACCCUGACUAUAAGUACA
antisense	UGUACUUAUAGUCAGGGUACUUCUC
siSOX5-3	sense	CAAGACAGCAGCAGCAGCTTCTACA-3′
antisense	TGTAGAAGCTGCTGCTGCTGTCTTG
siDNMT1-1	sense	GCCUCAUCGAGAAGAAUAUTT
antisense	AUAUUCUUCUCGAUGAGGCTT
siDNMT1-2	sense	GGGACUGUGUCUCUGUUAUTT
antisense	AUAACAGAGACACAGUCCCTT
siDNMT1-3	sense	CAGTCCCGAGTATGCGCCCATATTT
antisense	AAATATGGGCGCATACTCGGGACTG
sip21-1	sense	GCGAUGGAACUUCGACUUUTT
antisense	AAAGUCGAAGUUCCAUCGCTT
sip21-2	sense	GAUGGAACUUCGACUUUGUTT
antisense	ACAAAGUCGAAGUUCCAUCTT
sip21-3	sense	GACCAUGUGGACCUGUCACTT
antisense	GUGACAGGUCCACAUGGUCTT
siNC	sense	UUCUCCGAACGUGUCACGUTT
antisense	ACGUGACACGUUCGGAGAATT

The sequences of *SOX5*, *DNMT1* and *p21* were synthesized by Generay (Shanghai, China) and inserted into the pCDNA3.1 vector. The plasmids were transfected into the cells using Lipofectamine-2000 (Invitrogen, Carlsbad, USA) according to the manufacturer’s instructions. After 6 h of transfection, the medium containing the transfection reagent was replaced with fresh medium. Forty-eight hours after transfection, the cells were collected for further study.

### Quantitative real-time polymerase chain reaction (qRT-PCR)

Liquid nitrogen was used to grind the tissue samples. Trizol reagent (Junxin Biotech, Suzhou, China) was used to isolate the total RNA from tissues specimens or cells according to the manufacturer’s instructions. PrimeScriptTM RT Master Mix (TaKaRa, Dalian, China) was applied to perform the reverse transcription according to the protocols. SYBR Green I qPCR Mix (Junxin Biotech) was used for quantitative real-time PCR with the ABI 7500 real-time PCR system (Applied Biosystems, Foster City, USA). The 2 ^–ΔΔCT^ method was introduced to calculate the relative expression levels of genes which were normalized to that of *β-actin*. Primers used for real-time PCR were designed using the US National Center for Biotechnology Information (NCBI) ( http://www.ncbi.nlm.nih.gov/) and synthesized by Sangon Biotech. The sequences of these primers are shown in Table 3.

**Table:** **Table 3** Sequence of primers used for qRT-PCR

Gene	Primer sequence (5′→3′)
*h-SOX5*	Forward	CGATCATAGGTGGCTGCTGT
Reverse	5ATAGCTGAAGCCTGGAGGGA
*h-DNMT1*	Forward	AGGGCTACCTGGCTAAAGTC
Reverse	CCTCTCCATCGGACTTGCTC
*h-p21*	Forward	GGCCACAGGCCAGCTTCCA
Reverse	TGTGCACAACACCTGTGTC
*h-β-Actin*	Forward	CATTCCAAATATGAGATGCGTTGT
Reverse	TGTGGACTTGGGAGAGGACT
*SOX5*	Forward	AGGTTTGGACTCACTTGACAGG
Reverse	GTGAGGCTTGTTGGGAAAACTC
*DNMT1*	Forward	AGGGCTACCTGGCTAAAGTC
Reverse	CCTCTCCATCGGACTTGCTC
*p21*	Forward	TGTCCGTCAGAACCCATGC
Reverse	AAAGTCGAAGTTCCATCGCTC
*β-Actin*	Forward	ATGGATGACGATATCGCTGC
Reverse	CTTCTGACCCATACCCACCA

### Western blot analysis

The clinical specimens were ground with liquid nitrogen. Then, RIPA lysis buffer was introduced to extract the proteins from clinical specimens and cells. The protein concentration was quantified using a BCA detection kit (Junxin Biotech). Protein extract (30 μg) was separated by sodium dodecyl sulphate-polyacrylamide gel electrophoresis (SDS-PAGE) and electrophoretically transferred onto a nitrocellulose membrane (Millipore, Billerica, USA). Membranes were blocked with 5% skim milk, incubated with primary antibodies and horseradish peroxidase (HRP)-labeled secondary antibody successively. The protein bands were visualized using an ECL reagent (Junxin Biotech) on Gel Imagine System (Bio-Rad, Hercules, USA). Antibodies used in this study are shown as follows: anti-DNMT1 (Abcam, Cambridge, UK; ab87656), anti-SOX5 (Millipore; 07e131) and anti-p21 (Abcam; ab109520). The secondary antibody was purchased from Service Biotechnology Co., Ltd (Wuhan, China). The dilution ratio of antibodies was 1:1000.

### Cell viability assay

Cell Counting Kit-8 (CCK8; Jinxin Biotech) was used to determine cell viability according to the manufacturer’s instructions. For CCK8 analysis, the cells were planted into 96-well plates (4×10 ^3^ cells/well). After different treatments, the cells in each well were added with 10 μL of CCK8 regent. After incubation at 37°C for 2 h, absorbance was detected at OD 450 nm using a micro-plate reader (Thermo Scientific, Waltham, USA).

### Cell proliferation assay

The 5-ethynyl-2′-deoxyuridine (EdU) kit (Junxin Biotech) was used to determine cell proliferation according to the manufacturer’s instructions. Before the EdU analysis, the cells were changed with fresh medium containing 200 μL of EdU regent, and grew at 37°C for 2 h. After being washed with 1 ×PBS buffer, the cells were fixed with 4% paraformaldehyde (PFA). Then, the cells were permeabilized with PBS containing 0.3% Triton X-100. Ultimately, the cells were stained with Apollo and Hoechst successively. The stained cells were observed under a fluorescence microscope (ZKX53; Olympus, Tokyo, Japan). Red signals were EdU-stained cells, representing proliferating cells and meanwhile, and the blue signal was the nucleus, representing the overall cell number.

### Cell migration assay

The Transwell chamber (Corning, Corning, New York, USA) was used to measure the migration ability of cells according to the manufacturer’s instructions. Briefly, the cells were suspended in medium supplemented with 1% FBS before being seeded into the upper chamber (10,000 cells/well), and then the insert was added to the 24-well plate which was filled with 600 μL medium supplemented with 10% FBS. Twenty-four hours later, the cells in the chamber were removed with cotton swabs, and the migrated cells in the outer membrane of the chamber were stained with 0.1% purple crystal at room temperature for 30 min. After the air-drying, the migrated cells were observed under a microscope (ZKX53) and the number of migrated cells was counted.

### Cell apoptosis assay

Apoptosis in NRCMs was determined by a TdT-mediated dUTP Nick-End Labeling (TUNEL) kit (Roche, Basel, Switzerland) based on the operation instructions. After being washed with 1 ×PBS buffer, the cells were fixed with 4% paraformaldehyde (PFA) and permeabilized with PBS containing 0.3% Triton X-100. Then, the cells were incubated with 50 μL TUNEL detection regents at 37°C for 1 h in the dark, and stained with Hoechst solution for 15 min at 37°C in the dark. The stained cells were observed under a fluorescence microscope (ZKX53). Green signals were TUNEL-stained cells, representing apoptotic cells, and meanwhile, the blue signal was the nucleus, representing the overall cell number.

### Methylation-specific PCR (MSP) analysis

The Genomic DNA was extracted using the Genomic DNA Mini Preparation kit with Spin Column (Beyotime, Shanghai, China) according to the manufacturer’s protocol. Genomic DNA was modified by EZ DNA methylation-GOLD kit (ZYMO Research, Irvine, USA) according to the manufacturer’s instructions. The primers for the MSP are listed in Supplementary File 1.

### Chromatin Immunoprecipitation (ChIP) assay

ChIP analysis was carried out according to the previously described method [21]. The antibodies used in ChIP analysis were listed below: anti-DNMT1 (Abcam; ab87656), and anti-SOX5 (Millipore; 07e131). The DNA of ChIP samples was isolated with the Genomic DNA Mini Preparation kit with Spin Column (Beyotime) according to the manufacturer’s protocol, and the enrichment of DNMT1 promoter was measured using quantitative real-time PCR. The primers for DNMT1 promoter were: DNMT1-promoter F: 5′-CCCCCACACACTGGGTATAG-3′ and DNMT1-promoter R: 5′-AGGGTTTGTGAGAGCCCTTG-3′.

### Generation of xenografts

Five week-old female athymic nude mice were obtained from the Model Animal Research Institute of Nanjing University (Nanjing, China). The right flanks of mice were injected subcutaneously with T24 cells (1×10 ^7^ cells in 0.2 mL sterile PBS). The volume of xenografts was measured every week using calipers and calculated as follows: tumor volume=(length×width ^2^)/2. The mice were euthanized one month after injection, and the xenografts were collected for further analyses. All animal experimental procedures were approved by the Committee on the Ethics of Animal Experiments of the Xiangya Hospital, Central South University.

### Statistical analysis

Experiments were performed in triplicate and data were analyzed using GraphPad Prism 5.0 (GraphPad Software Inc., La Jolla, USA) and expressed as the mean±SD. Student’s *t*-test was applied to assess the statistical differences. *P*<0.05 represents a statistically significant difference.

## Results

### SOX5 is up-regulated in BC tissues and *in vitro* cell lines

**Figure 1:**
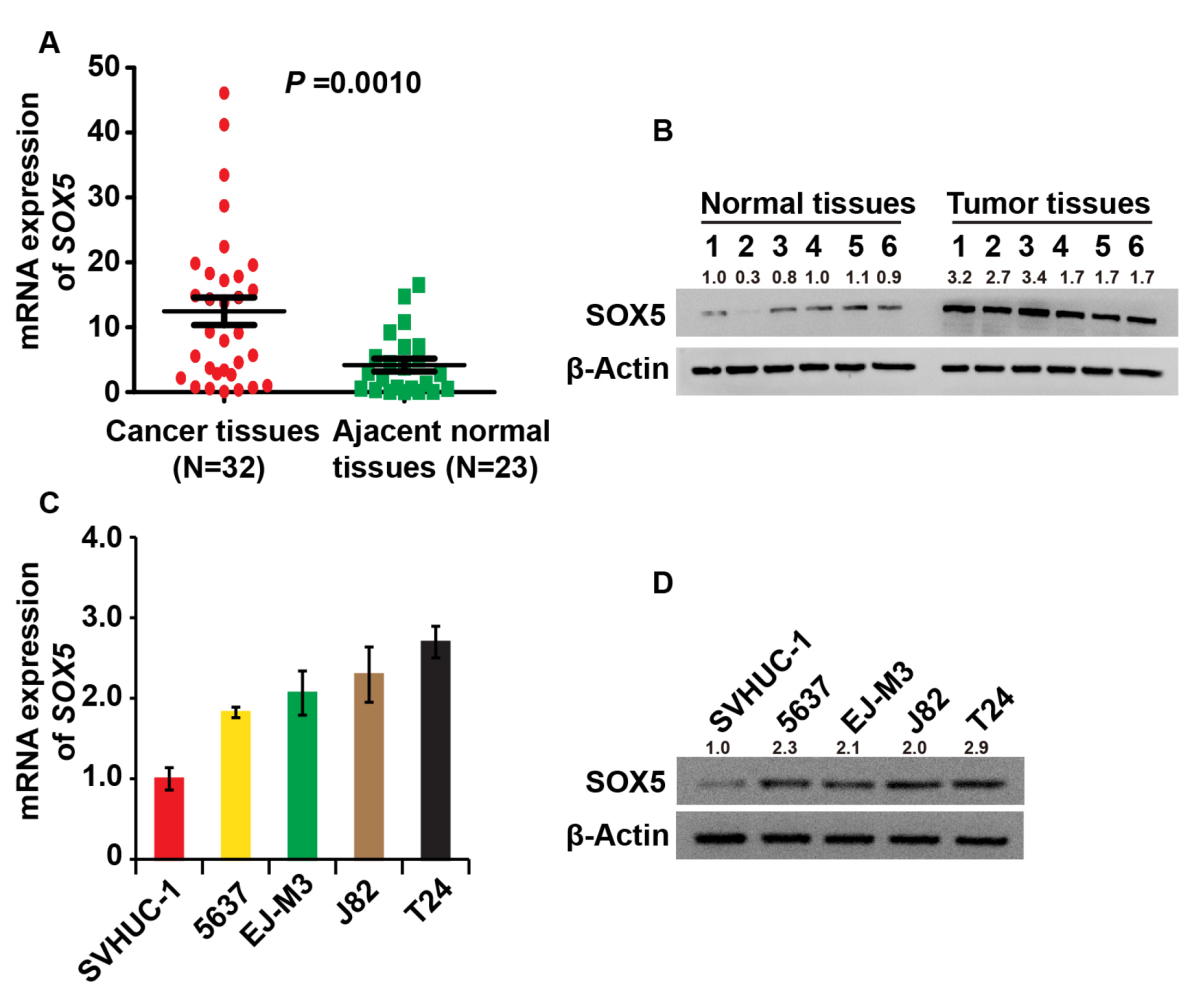
Figure 1 SOX5 is up-regulated in BC tissues and *in vitro* cell lines (A,B) The expression of SOX5 was measured in clinical BC ( n=32) and adjacent normal tissues ( n=23) by qPCR and western blot analysis. (C,D) The expression of SOX5 was detected in different cell lines by qPCR and western blot analysis. The cells consisted of BC cell lines 5673, EJ-M3, J82, T24, and immortalized human bladder epithelial SVHUC-1 cells. β-Actin was used as the loading control. Experiments were repeated three times.

To investigate the role of SOX5 in BC, human BC tissues as well as paired adjacent norm tissues were collected, and qPCR and western blot analysis were used to measure the SOX5 expression in these tissues. The results showed that SOX5 expression in BC was higher than that in normal tissues, both at the RNA and protein levels ( Figure 1A,B).

Then SOX5 expressions in human BC cell lines 5673, J82, T24, EJ-M3, and SVHUC-1 bladder epithelial cells were determined. Results showed that SOX5 expression was elevated in BC cell lines, as it was in BC tissues, when compared to bladder epithelial cells ( Figure 1C, D). These findings suggest that SOX5 may play a role in the progression of BC.

### SOX5 positively regulates the cell process of BC cells

**Figure 2:**
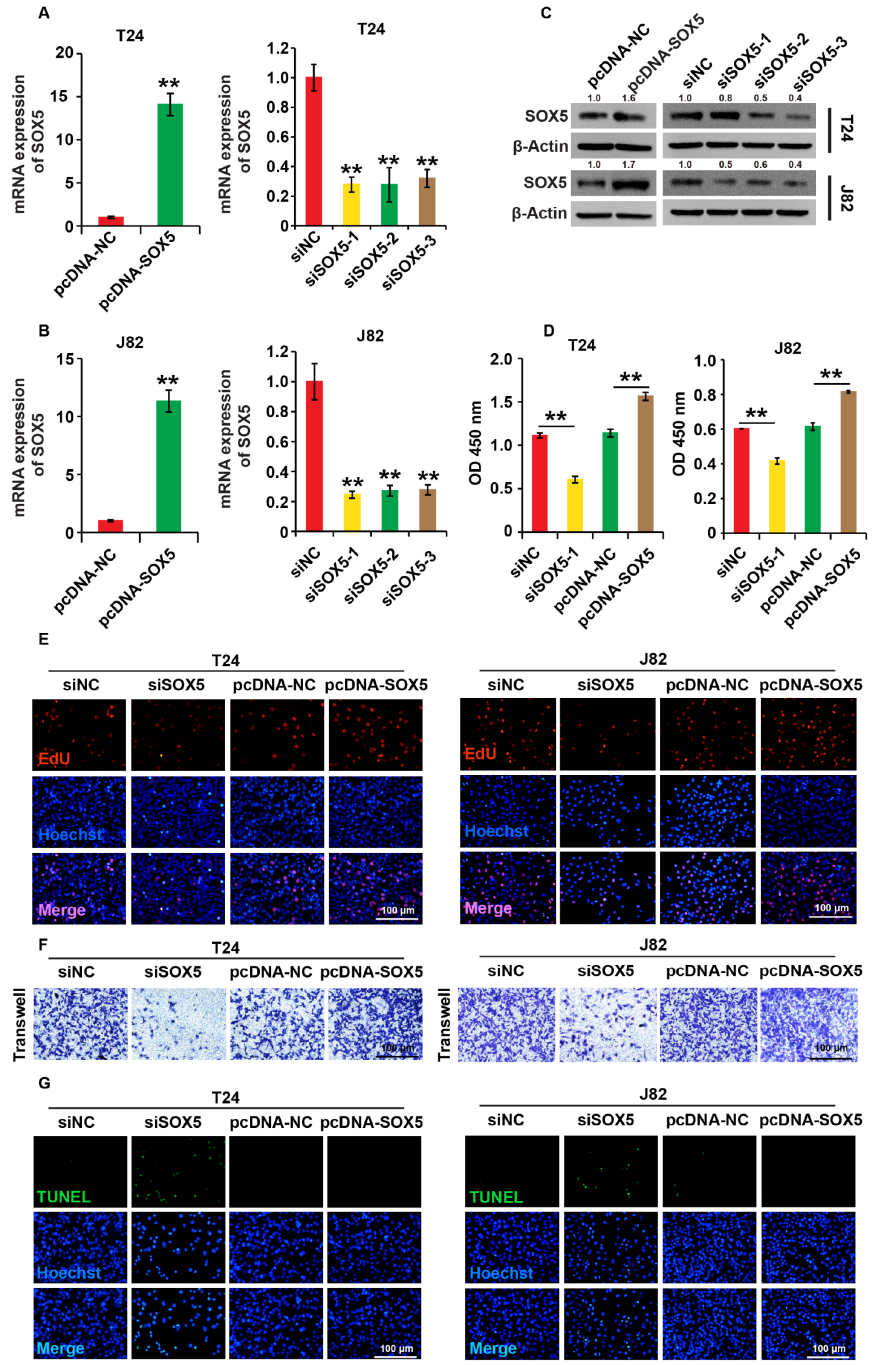
Figure 2 SOX5 positively regulates the cellular processes of BC cells (A,B) The expression of SOX5 was measured by qPCR analysis in T24 and J82 cells. (C) The expression of SOX5 was measured by western blot analysis in T24 and J82 cells. (D) The cell viability was measured by CCK8 assay. (E) The cell proliferation was determined by EdU assay. (F) The cell migration was detected by Transwell assay. (G) The cell apoptosis was measured using the TUNEL assay. Cells were transfected with pcDNA-SOX5, pcDNA-NC, SOX5-siRNAs (siSOX5) and control siRNAs (siNC), respectively. β-Actin was used as the loading control. ** P<0.01. Experiments were repeated three times.

To investigate the role of SOX5 in cancer progression, we first created a SOX5 overexpression plasmid (pcDNA-SOX5) based on the pcDNA-3.1 vector, then synthesized siRNAs against SOX5 (SOX5-siRNAs), and transfected them into T24 and J82 cells. First, we used qPCR and western blot analysis to detect SOX5 mRNA and protein expression levels respectively and discovered that pcDNA-SOX5 significantly increased SOX5 expression, while SOX5-siRNAs significantly inhibited its expression in both cell lines ( Figure 2A–C). In the subsequent experiments, only the results of siSOX5-1 were shown. The cell viability, proliferation, migration, and apoptosis of BC cells were investigated by the CCK8, EdU, Transwell, and TUNEL assays, respectively. SOX5 overexpression increased cell viability, proliferation, and migration in both T24 and J82 cells. Meanwhile, SOX5 knockdown had the opposite effect on the cellular processes of BC cells ( Figure 2D–F and Supplementary Figure S1A,B). siSOX5 induced apoptosis in BC cells, but no differences in apoptosis were found between the pcDNA-NC and pcDNA-SOX5 groups ( Figure 2G and Supplementary Figure S1C). All these results revealed that the knockdown of SOX5 can inhibit the cell viability, proliferation and migration, and it can also promote apoptosis of BC cells.

### SOX5 promotes DNMT1 expression in BC cells

**Figure 3:**
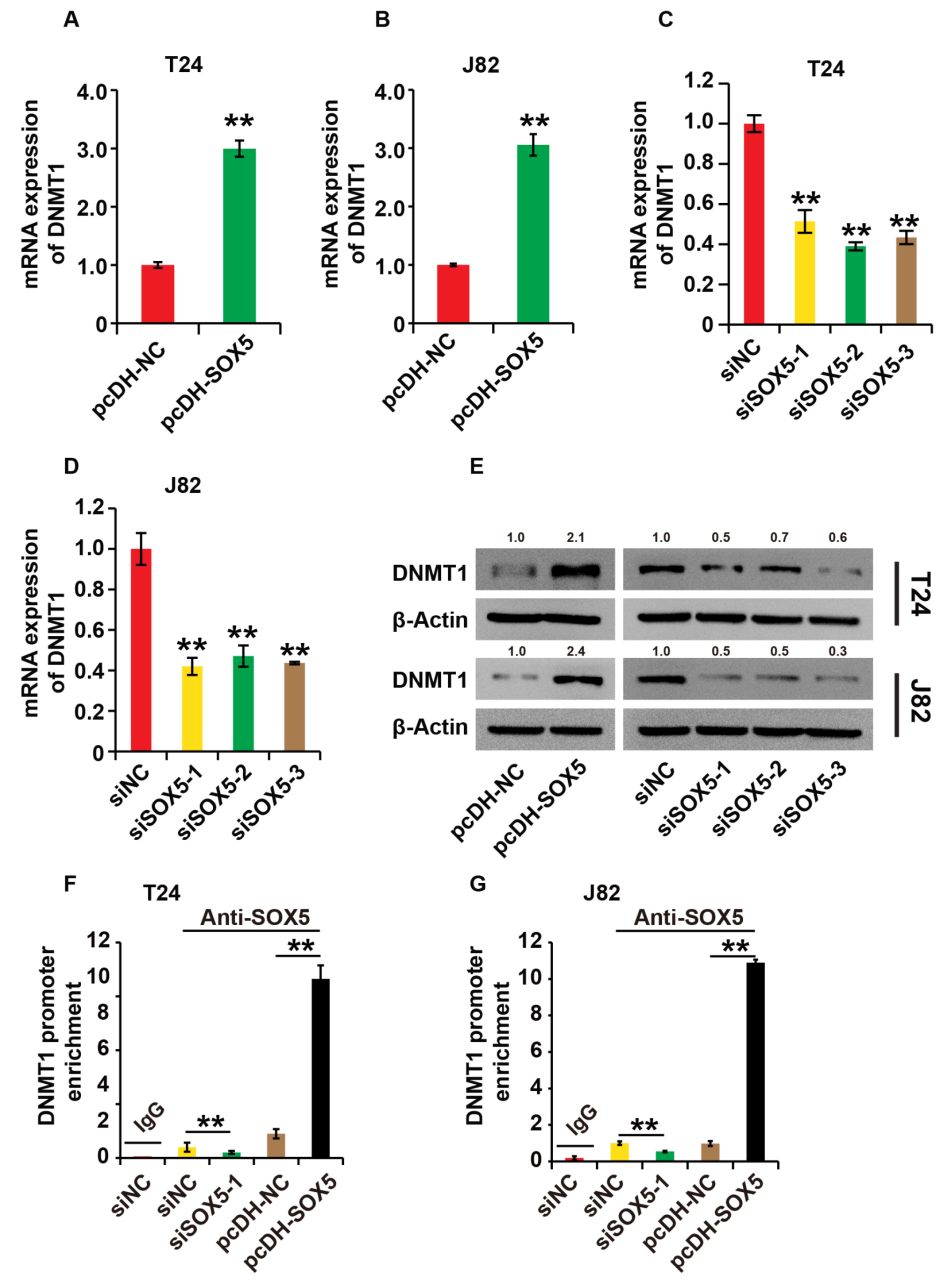
Figure 3 SOX5 promotes DNMT1 expression in BC cells (A,B) The expression of SOX5 was measured by qPCR analysis in T24 and J82 cells transfected with pcDNA-SOX5 and pcDNA-NC, respectively. (C,D) The expression of SOX5 was measured by qPCR analysis in T24 and J82 cells transfected with siSOX5 and siNC, respectively. (E) The expression of SOX5 was measuered by western blot analysis in T24 and J82 cells. (F,G) The enrichment of the DNMT1 promoter was determined by ChIP analysis in T24 and J82 cells. For (E–G), cells were transfected with pcDNA-SOX5, pcDNA-NC, siSOX5 and siNC, respectively. β-Actin was used as the loading control. ** P<0.01. Experiments were repeated three times.

Because DNMT1 has been linked to BC [22], we wanted to know if SOX5 could influence DNMT1 expression in BC cells. We first transfected BC cells with pcDNA-SOX5, pcDNA-NC, SOX5-siRNAs, and NC-siRNAs. We detected DNMT1 expression after 48 h, and the results showed an increased DNMT1 expression in cells transfected with pcDNA-SOX5, compared to cells transfected with pcDH-NC ( Figure 3A,B,E), and an inversely decreased DNMT1 expression in cells transfected with SOX5-siRNAs, compared to cells transfected with NC-siRNAs ( Figure 3C–E). ChIP analysis revealed that SOX5 could bind to the DNMT1 promoter, indicating that SOX5 regulates DNMT1 expression at the transcriptional level ( Figure 3F,G). These findings demonstrate that SOX5 increases DNMT1 expression in BC cells.

### Ectopic expression of DNMT1 rescues the cellular processes caused by SOX5 knockdown in BC cells

**Figure 4:**
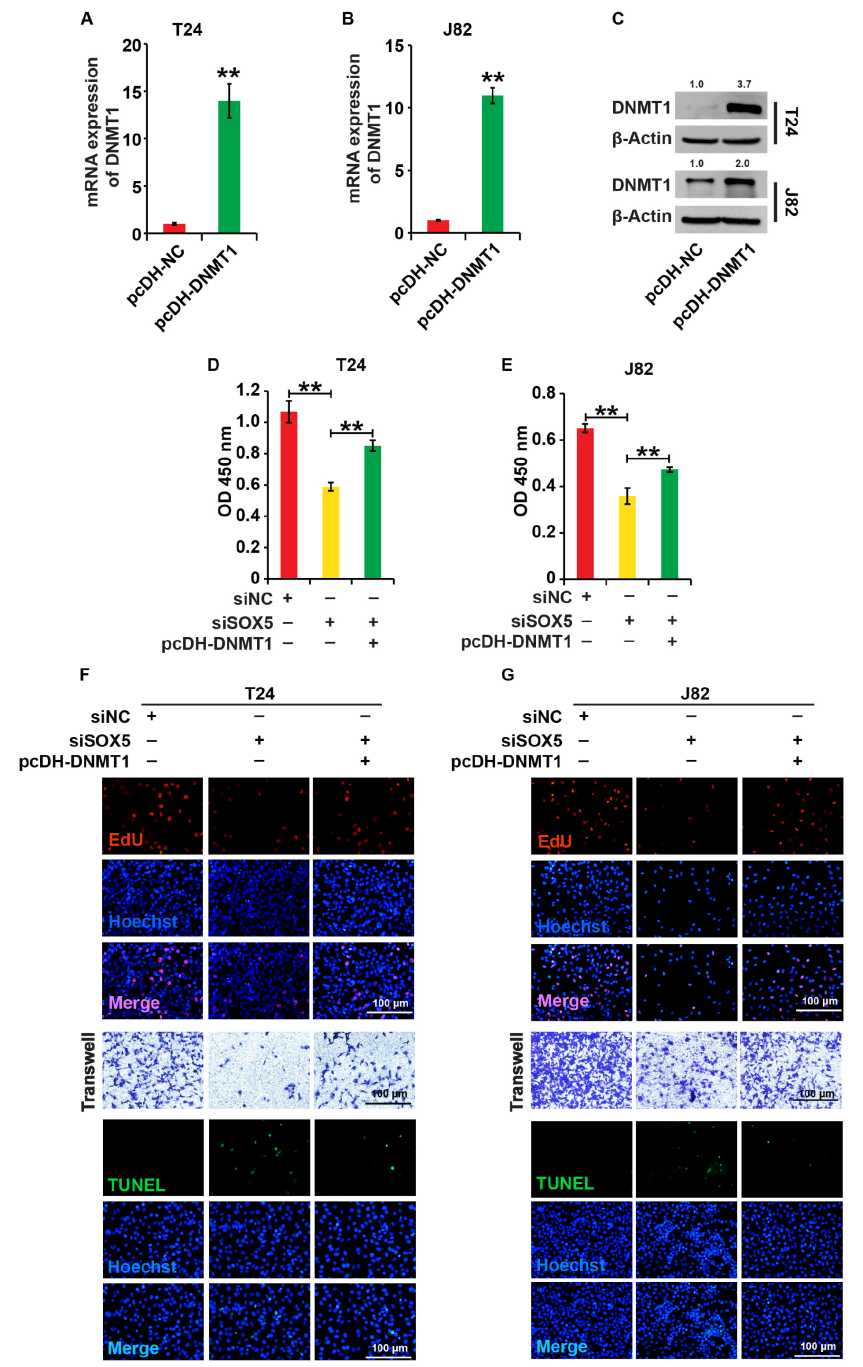
Figure 4 Ectopic expression of DNMT1 rescues the cell processes caused by SOX5 knockdown in BC cells (A–C) The expression of DNMT1 was measured by qPCR and western blot analysis in T24 and J82 cells transfected with pcDNA-DNMT1 and pcDNA-NC, respectivel. (D,E) The cell viability was measured by the CCK8 assay. (F,G) The cell proliferation, migration and apoptosis were measured by EdU assay, Transwell assay and TUNEL assay, respectively. The cells were divided into three groups: pcDNA-DNMT1+siSOX5, siSOX5 and siNC. β-Actin was used as the loading control. ** P<0.01. Experiments were repeated three times.

We overexpressed DNMT1 (pcDNA-DNMT1) in wild-type cells or in SOX5-knockdown T24 and J82 cells to demonstrate if it mediates SOX5’s role in BC cells. qPCR and western blot analysis revealed that pcDNA-DNMT1 significantly increased DNMT1 expression in wild-type cells ( Figure 4A–C). CCK8, EdU, Transwell, and TUNEL analysis revealed that DNMT1 overexpression reversed cellular processes in BC cells caused by SOX5 knockdown ( Figure 4D–G and Supplementary Figure S1D–F), implying that SOX5’s role in BC cells is mediated by DNMT1.

### DNMT1 inhibits p21 expression in BC cells

**Figure 5:**
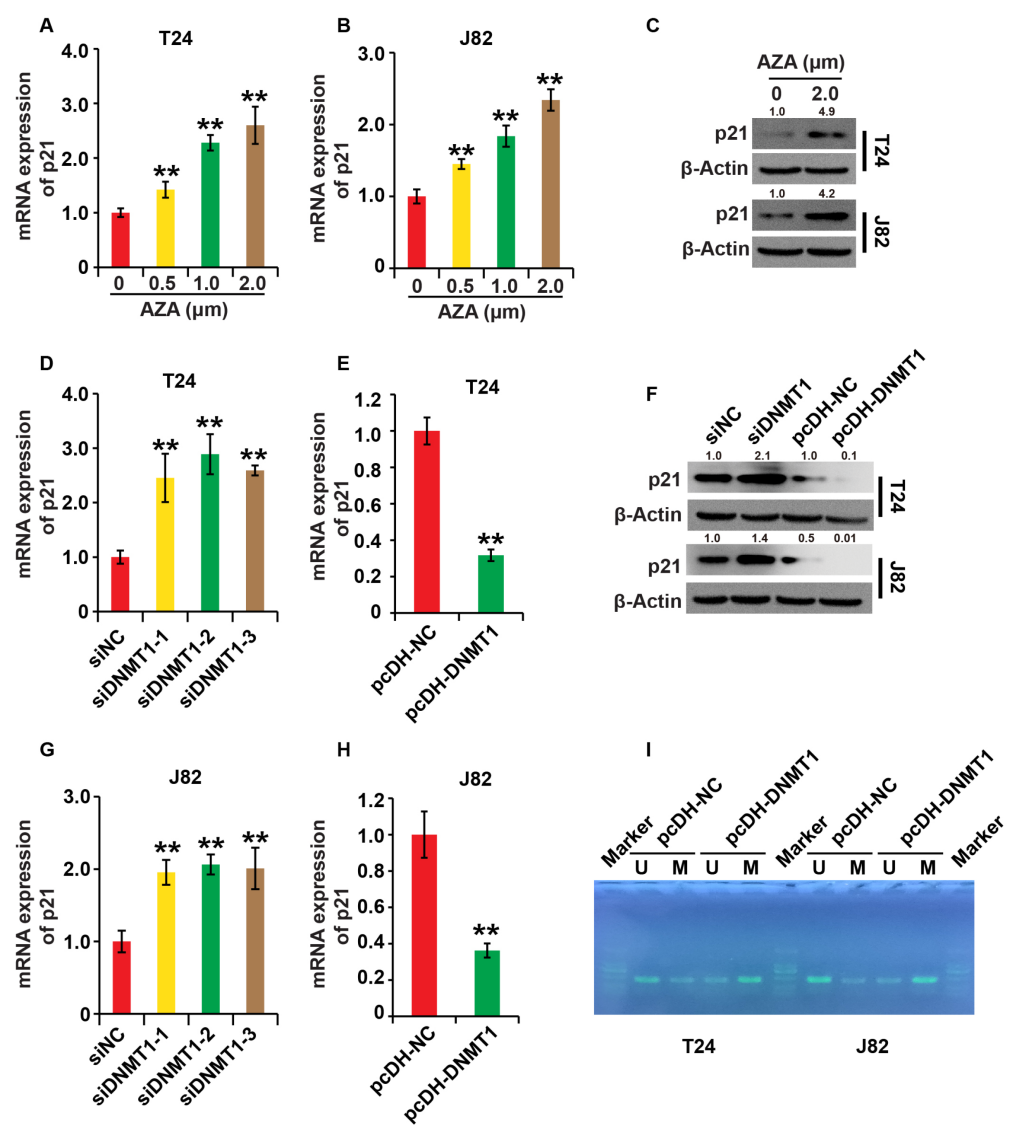
Figure 5 DNMT1 inhibits p21 expression in BC cells (A,B) The expression of p21 was measured by qPCR analysis in T24 and J82 cells treated with 5-AzadC at different concentrations (0, 0.5, 1.0, 2.0 μM) for 24 h. (C) The expression of p21 was measured by western blot analysis in T24 and J82 cells treated with 5-AzadC (0, 2.0 μM) for 24 h. (D–H) The expression of p21 was measured by qPCR and western blot analysis in T24 and J82 cells transfected with pcDH-DNMT1, pcDH-NC, siDNMT1 and siNC, respectively. (I) The DNA methylation stage of the p21 promoter was measured by MSP analysis in T24 and J82 cells transfected with pcDH-DNMT1 and pcDH-NC, respectively. β-Actin was used as the loading control. ** P<0.01. Experiments were repeated three times.

It was reported that DNMT1-mediated DNA methylation regulated p21 expression in cancer cells [23]. To investigate the regulatory relationships between DNMT1 and p21 in BC cells, we treated the cells for 24 h with 5-AzadC at various concentrations (0, 0.5, 1.0, and 2.0 μM). qPCR and western blot analysis revealed that 5-AzadC significantly increased p21 expression in both cell lines ( Figure 5A–C). Then, in BC cells, we knocked down and overexpressed DNMT1. As expected, pcDNA-DNMT1 decreased p21 expression in both cells, whereas DNMT1-siRNAs significantly increased its expression ( Figure 5D–H). Methylation-specific PCR analysis revealed that pcDNA-DNMT1 increased the DNA methylation stage of the p21 promoter, whereas DNMT1-siRNAs inhibited the DNA methylation stage of the p21 promoter ( Figure 5I). These findings demonstrate that DNMT1 inhibits p21 expression in BC cells.

### SOX5/DNMT1/p21 pathway regulates cell processes of BC cells

**Figure 6:**
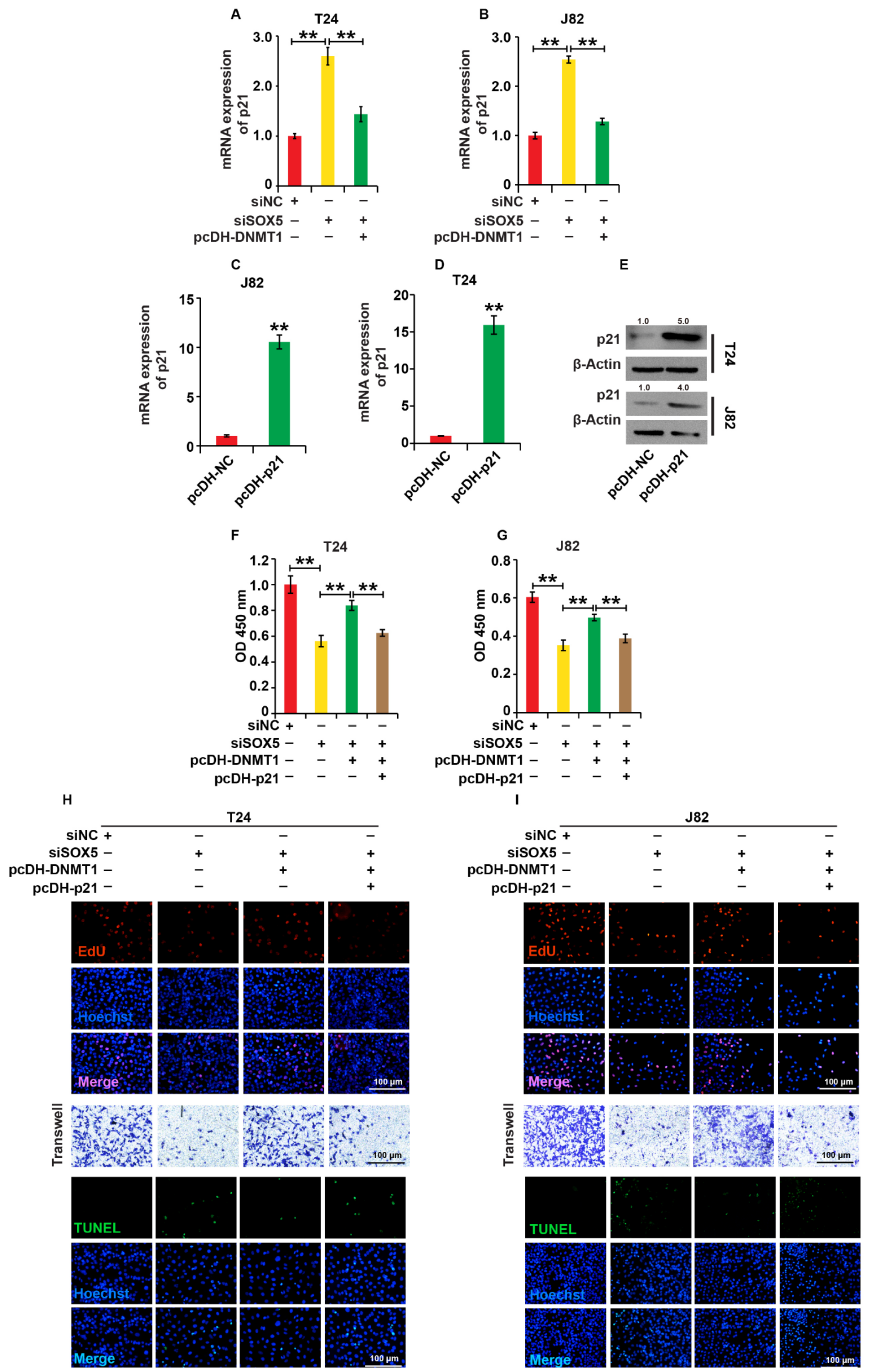
Figure 6 SOX5/DNMT1/p21 pathway regulates cell processes of BC cells (A,B) The expression of p21 was measured by qPCR analysis in T24 and J82 cells. The cells were divided into four groups: pcDH-DNMT1+siSOX5+pcDH-p21, pcDH-DNMT1+siSOX5, siSOX5 and siNC. (C–E) The expression of p21 was measured by western blot analysis in T24 and J82 cells transfected with pcDH-p21 and pcDH-NC, respectively. (F,G) The cell viability was measured by the CCK8 assay. (H,I) The cell proliferation, migration and apoptosis were measured by EdU assay, Transwell assay and TUNEL assay, respectively. For (F–I), the cells were divided into four groups: pcDNA-p21+pcDNA-DNMT1+siSOX5, pcDNA-DNMT1+siSOX5, siSOX5 and siNC. β-Actin was used as the loading control. ** P<0.01. Experiments were repeated three times.

We used three groups of cells, *i*. *e*., pcDH-DNMT1+siSOX5, pcDH-NC+siSOX5, and pcDH-NC+siNC, to investigate the upstream and downstream regulatory relationship between DNMT1 and p21 in the function of SOX5 in BC. qPCR and western blot analysis revealed that pcDNA-DNMT1 reversed the up-regulation of p21 expression caused by SOX5-siRNAs in both cells ( Figure 6A,B), indicating that SOX5 silencing increased p21 expression by down-regulating DNMT1 expression.

To investigate the role of the SOX5/DNMT1/p21 pathway in the cellular processes of BC cells, we first constructed a p21 overexpression vector (pcDH-p21) based on the pcDNA-3.1 vector and transfected it into T24 and J82 cells qPCR and western blot analysis revealed that pcDH-p21 significantly increased p21 expression in both cell lines ( Figure 6C–E). Then, we performed experiments on four different groups of cells, *i*. *e*., pcDH-p21+pcDH-DNMT1+siSOX5-siRNAs, pcDH-DNMT1+siSOX5, pcDH-NC+siSOX5, and pcDH-NC+siNC. CCK8, EdU, Transwell, and TUNEL analysis revealed that any disruption of the SOX5/DNMT1/p21 pathway impacted cellular processes of BC cells ( Figure 6F–I and Supplementary Figure S1G–I). These results demonstrate that SOX5 regulates cellular processes in BC cells via the SOX5/DNMT1/p21 pathway.

### Knockdown of SOX5 suppresses the growth of xenograft tumors *in vivo*

**Figure 7:**
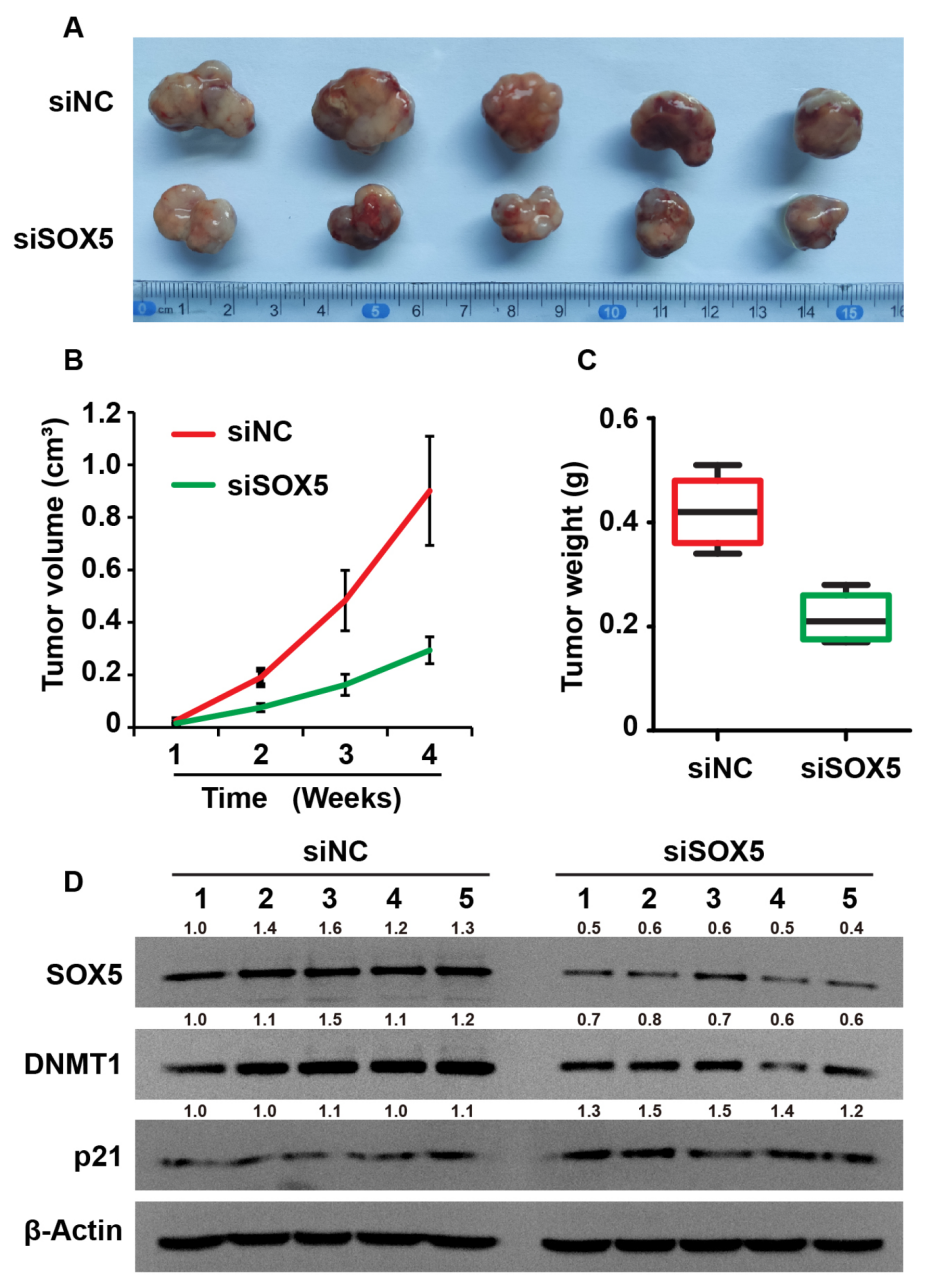
Figure 7 Knockdown of SOX5 suppresses the growth of xenograft tumors *in vivo* (A) Representative images of the xenograft tumors in BALB/c nude mice. (B) The growth curves of xenograft tumors at different time points. (C) The relative weights of tumors were determined at different time points. (D) Western blot analysis of Sox5, DNMT1 and p21 expressions in xenograft tumors and tissues of BALB/c nude mice. The T24 cells with stable SOX5 knockdown were used to induce the formation of the xenograft tumors in BALB/c nude mice. β-Actin was used as the loading control. There were five mice in each group.

Given the importance of SOX5 in BC cells, we investigated whether SOX5 affects the growth of xenograft tumors *in vivo*. T24 cells with stable knockdown of *SOX5* were intraperitoneally injected into mice. The results showed that knockdown of *SOX5* inhibited the growth of xenograft tumors *in vivo* ( Figure 7A–C). Furthermore, the expressions of SOX5 and DNMT1 were reduced, while p21 expression was increased in xenograft tumors treated with siSOX5 ( Figure 7D). These findings confirm that suppressing SOX5 inhibits the growth of xenograft tumors *in vivo*.

## Discussion

BC is a common urological tumor and one of the most common male malignant tumors. Despite advances in diagnosis, treatment and experience, the 5-year survival rate for BC remains very low [24]. A better understanding of tumor characteristics aids in the development of new diagnostic and therapeutic approaches. The hallmarks of tumors are summarized by Douglas Hanahan and Robert A. Weinberg, which include self-sufficiency in growth signals, insensitivity to antigrowth signals, resistance to cell death, limitless replicative potential, sustained angiogenesis, tissue invasion and metastasis, avoiding immune destruction, tumor promotion inflammation, deregulating cellular energetics, genome instability, and mutation [ 25, 26] . As a result, the essence of tumor prevention and treatment is to inhibit these ten tumor characteristics. Naltrexone can activate the PI3K/AKT signaling pathway in BC [27]. LINC00355 was discovered to promote BC cell proliferation, invasion, and resistance to cisplatin [ 28, 29] . All of these previous studies have revealed potential targets for promoting BC development. In our study, we discovered that the SOX5/DNMT1/p21 pathway plays an important role in the cellular processes of BC, such as cell viability, proliferation and migration, indicating the potential application of this pathway in the diagnosis and treatment of BC.

In vertebrates, the SOX family contains more than 20 genes divided into eight groups [5]. The SOX family, as an important group of transcription factors, plays important roles in cell growth and apoptosis, cell cycle, metastasis, epithelial to mesenchymal transition (EMT), metabolism, and tumorigenesis’ physiological and pathological progression [5]. SOX5 has been linked to breast cancer, gastric cancer, colorectal cancer, lung cancer, and osteosarcoma [ 30– 34] . SOX5 also regulates a variety of targets in cancers, including EZH2, twist 1, P2RY8, MITF, and others [ 6, 7, 35, 36] .

However, there has not been a lot of research done in BC on the function of SOX5 and its underlying molecular mechanism. SOX5 was found to be up-regulated in BC in our study. Further research revealed that *SOX5* knockdown inhibited cell growth and migration in BC cells, whereas SOX5 overexpression promoted these cellular processes. Furthermore, silencing of *SOX5* inhibited tumor growth *in vivo* . These findings provide preliminary support for SOX5’s oncogenic role in BC.

DNA methylation is a common mode of gene expression regulation. As the primary DNA methylation transferase, DNMT1 plays a critical role in tumorigenesis. The oncogenic role of DNMT1 in a variety of cancers, including BC, has received considerable attention [37]. Nonetheless, no research has been conducted to demonstrate the regulatory relationships between SOX5 and DNMT1 in BC. Therefore, we investigated whether SOX5 plays a role in BC by regulating DNMT1. Our findings showed that SOX5 promoted DNMT1 expression, and that ectopic expression of DNMT1 reduced the role of inhibited SOX5 in the cellular functions of BC cells.

DNMT1 inhibits tumor suppressor genes in several cancers by hyper-methylating gene promoters [38]. Sanaei et al. [39] discovered that DNMT1 repression increased p21 expression in the colon cancer SW480 cell line. Statins, according to Dongoran *et al*. [40], had an anti-proliferative effect in OSCC cells by inhibiting DNMT1-regulated p21 expression. As a result, we are curious to know if DNMT1 regulates p21 in BC. Our findings first confirmed DNMT1’s inhibitory regulation of p21 in BC cells. More excitingly, overexpression of DNMT1 reversed the elevation of p21 caused by silenced *SOX5*, implying that SOX5 silencing down-regulates DNMT1 expression, leading to the elevation of p21 expression in BC cells.

In conclusion, we elucidated the role and molecular mechanism of SOX5 in BC. Our findings showed that SOX5 expression was increased in BC tissues and *in vitro* cell lines, confirming SOX5’s oncogenic role in BC. Our study revealed that increased SOX5 expression promoted DNMT1 expression and inhibited p21 expression via DNA methylation, indicating a potential role for the SOX5/DNMT1/p21 pathway in the diagnosis and treatment of BC.

## Supporting information

21496Tables

## Supplementary Data

Supplementary data is available at *Acta Biochimica et Biophysica Sinica* online.
