# Supplementary material for: SOX5 promotes cell growth and migration through modulating DNMT1/p21 pathway in bladder cancer: SOX5/DNMT1/p21 pathway in BC
Source: Acta Biochim Biophys Sin (Shanghai). 2022 Jun 21;54(7):987–98. doi: 10.3724/abbs.2022075 (PMC9909322; doi:10.3724/abbs.2022075)
Supplement: 21496Tables [file 21496Tables.pdf]

**Table 1. Clinical information of BC patients**

| Characteristics | Age | | Gender | | T stage | | N stage | | M stage | | Grade | |
|---|---|---|---|---|---|---|---|---|---|---|---|---|
| | ≤65 | > 65 | Male | Female | Ta–T1 | T2–T4 | N– | N+ | M- | M+ | High | Low |
| Number | 24 | 8 | 31 | 1 | 14 | 18 | 27 | 5 | 32 | 0 | 25 | 7 |

**Table 2. The sequences of siRNAs used in the study**

| Name | Sequence (5′→3′) | |
|---|---|---|
| siSOX5-1 | sense | GACCAUGAUGCUGUCACCAAGGCAA |
| | antisense | UUGCCUUGGUGACAGCAUCAUGGUC |
| siSOX5-2 | sense | GAGAAGUACCCUGACUAUAAGUACA |
| | antisense | UGUACUUAUAGUCAGGGUACUUCUC |
| siSOX5-3 | sense | CAAGACAGCAGCAGCAGCTTCTACA-3' |
| | antisense | TGTAGAAGCTGCTGCTGCTGTCTTG |
| siDNMT1-1 | sense | GCCUCAUCGAGAAGAAUAUTT |
| | antisense | AUAUUCUUCUCGAUGAGGCTT |
| siDNMT1-2 | sense | GGGACUGUGUCUCUGUUAUTT |
| | antisense | AUAACAGAGACACAGUCCCTT |
| siDNMT1-3 | sense | CAGTCCCGAGTATGCGCCCATATTT |
| | antisense | AAATATGGGCGCATACTCGGGACTG |
| sip21-1 | sense | GCGAUGGAACUUCGACUUUTT |
| | antisense | AAAGUCGAAGUUCCAUCGCTT |
| sip21-2 | sense | GAUGGAACUUCGACUUUGUTT |
| | antisense | ACAAAGUCGAAGUUCCAUCTT |
| sip21-3 | sense | GACCAUGUGGACCUGUCACTT |
| | antisense | GUGACAGGUCCACAUGGUCTT |
| siNC | sense | UUCUCCGAACGUGUCACGUTT |
| | antisense | ACGUGACACGUUCGGAGAATT |

## Table 3. Sequence of primers used for qRT-PCR

| Gene | Primer sequence (5′→3′) | |
|---|---|---|
| *h-Sox5* | Forward | CGATCATAGGTGGCTGCTGT |
| | Reverse | 5ATAGCTGAAGCCTGGAGGGA |
| *h-DNMT1* | Forward | AGGGCTACCTGGCTAAAGTC |
| | Reverse | CCTCTCCATCGGACTTGCTC |
| *h-p21* | Forward | GGCCACAGGCCAGCTTCCA |
| | Reverse | TGTGCACAACACCTGTGTC |
| *h-β-Actin* | Forward | CATTCCAAATATGAGATGCGTTGT |
| | Reverse | TGTGGACTTGGGAGAGGACT |
| *SOX5* | Forward | AGGTTTGGACTCACTTGACAGG |
| | Reverse | GTGAGGCTTGTTGGGAAAACTC |
| *DNMT1* | Forward | AGGGCTACCTGGCTAAAGTC |
| | Reverse | CCTCTCCATCGGACTTGCTC |
| *p21* | Forward | TGTCCGTCAGAACCCATGC |
| | Reverse | AAAGTCGAAGTTCCATCGCTC |
| *β-Actin* | Forward | ATGGATGACGATATCGCTGC |
| | Reverse | CTTCTGACCCATACCCACCA |
